# The Necessity of Taking Culture and Context into Account When Studying the Relationship between Socioeconomic Status and Brain Development

**DOI:** 10.3390/brainsci14040392

**Published:** 2024-04-18

**Authors:** Julie M. Schneider, Mohammad Hossein Behboudi, Mandy J. Maguire

**Affiliations:** 1 Department of Communication Sciences and Disorders, Louisiana State University, 72 Hatcher Hall, Field House Drive, Baton Rouge, LA 70803, USA; juschnei@lsu.edu; 2 Callier Center for Communication Disorders, The University of Texas at Dallas, 1966 Inwood Road, Dallas, TX 75235, USA; behboudi@utdallas.edu; 3 Center for Children and Families, The University of Texas at Dallas, 800 W Campbell Road, Dallas, TX 75080, USA

**Keywords:** socioeconomic status (SES), EEG, brain development, culture

## Abstract

Decades of research has revealed a relationship between childhood socioeconomic status (SES) and brain development at the structural and functional levels. Of particular note is the distinction between income and maternal education, two highly correlated factors which seem to influence brain development through distinct pathways. Specifically, while a families’ income-to-needs ratio is linked with physiological stress and household chaos, caregiver education influences the day-to-day language environment a child is exposed to. Variability in either one of these environmental experiences is related to subsequent brain development. While this work has the potential to inform public policies in a way that benefits children, it can also oversimplify complex factors, unjustly blame low-SES parents, and perpetuate a harmful deficit perspective. To counteract these shortcomings, researchers must consider sociodemographic differences in the broader cultural context that underlie SES-based differences in brain development. This review aims to address these issues by (a) identifying how sociodemographic mechanisms associated with SES influence the day-to-day experiences of children, in turn, impacting brain development, while (b) considering the broader cultural contexts that may differentially impact this relationship.

## 1. Introduction

### 1.1. Historical Perspectives Linking Socioeconomic Status (SES) and Brain Development

For decades, socioeconomic status (SES) was used as a covariate in studies of brain development in children, but starting around 2009, researchers became more interested in studying SES in and of itself as a predictor, or correlate, of differences in brain development [1]. This change was due to the understanding that SES can represent an individual’s access to various resources, which can directly impact brain development [2,3,4,5,6,7,8], educational attainment [9], physical well-being [10], mental health [11], and cognitive development [1]. As research has expanded to examine the impact of SES on brain development, researchers have acknowledged that SES is a complex construct, defined by correlated but not identical factors, such as parental educational attainment, occupational status, neighborhood quality, and family income. Recent work has made incredible strides in teasing apart the complexity of these relationships by studying both SES and brain structure and function from multiple perspectives. Through this work, researchers have begun to fill in gaps in our understanding of the complex relationship between an individual’s accumulated environmental experiences, their brain development, and how these differences in brain function and structure might manifest behaviorally. Despite these astounding efforts, most research examining the associations between SES and brain development are largely limited to WEIRD (Western, Educated, Industrial, Rich, and Democratic) countries, and often fail to consider the larger cultural context and belief systems that shape child cognitive and brain development. Here, we (1) review what is known about the relationship between SES and brain development based on magnetic resonance imaging (MRI) and resting state electroencephalography (EEG) studies; (2) pinpoint the unique influences of maternal education and income on this trajectory; and (3) review what questions remain to be answered, including taking a more in-depth, culturally responsive approach to understanding how a child’s day-to-day life experiences differentially relate to brain development.

### 1.2. The Importance of Studying the Association between SES and Brain Development

Brain maturation is a prolonged process that continues throughout childhood and adolescence. This extended period allows for a long window of experience-dependent plasticity, where early experiences can significantly shape brain development. During early development, the cortical thickness of the brain, linked to synaptic pruning and myelination, undergoes a rapid decrease before plateauing in early adulthood. Meanwhile, the surface area of the cortex undergoes a substantial expansion through early adolescence followed by a contraction through middle childhood [12,13,14]. Related to brain volume, white matter volume undergoes a rapid increase throughout adolescence and into young adulthood due to myelination before becoming reduced in older adulthood [15,16,17]. On the other hand, gray matter volume exhibits a rapid increase during early childhood, reflecting the growth and branching of dendrites and synaptogenesis before peaking in early adolescence, followed by a gradual volume thinning and density reduction due to synaptic pruning [15,16,18]. Studies using structural MRI have identified global differences in these developmental changes which are correlated to SES [1,6,7,19,20]. For example, coming from a lower SES background is often linked to less cortical gray matter, which researchers posit is due to less gray matter formation early in development, or accelerated thinning [5,20,21,22,23]. Meanwhile, children from higher SES backgrounds exhibit greater global and local efficiency of the structural connectome, indicative of enhanced neural communication speed and efficiency [24]. Specific areas of the brain, including the prefrontal cortex (PFC) [1,6,7,23,25,26,27], the left anterior and posterior superior temporal gyrus (STG) [28], and the left inferior frontal gyrus (IFG) [29,30], are sensitive to environmental experiences and support the development of language, attention, executive function, emotion regulation, and memory [5,31]. Thus, these differences in brain development have been the focus of much SES-related research. 

In addition to studies examining SES-related differences in brain *structure*, recent research has also sought to clarify SES-related differences in brain *function*. Specifically, SES has been linked to resting state brain function, as measured by both resting-state functional MRI (rsfMRI) and electroencephalography (rsEEG). rsfMRI and rsEEG allow for the investigation of spontaneous signal fluctuations in the brain when an individual is awake but not engaged in a specific cognitive task [32]. These spontaneous signal fluctuations are organized in multiple highly specific functional anatomical networks (resting state networks, RSNs) [33,34] which fluctuate at frequencies between 0.01 and 0.1 Hz, and strongly overlap with sensory–motor, visual, auditory, attention, language, and default networks [32,35]. These low-frequency fluctuations are thought to highly overlap between rsfMRI and rsEEG, as fluctuations in the band-limited power of the local field potential measured using EEG fluctuate at approximately the same frequency as the BOLD signal [36]. Importantly, both measures are thought to measure the brain’s “readiness” to predict and integrate new experiences, a critical component of cognitive development, as it allows children to generate and test predictions about their environment [37]. These measures are often used in diagnostic research, as they provide unique insights into brain development, related to changes in functional networks. 

Research utilizing rsfMRI has suggested that differences in functional brain development may be sensitive to neighborhood-and family-level-SES [38,39]. In these studies, coming from a lower SES background has been associated with reduced functional connectivity in the default mode network, sensorimotor network [40], and between the hippocampus and amygdala [41]. For example, children and adolescents living in more advantaged neighborhoods demonstrate a stronger positive association between age and functional connectivity compared to youth from more disadvantaged neighborhoods—a pattern which the authors interpret as being related to faster functional brain development among higher SES children [42]. Taken together, structural and functional fMRI studies suggest that coming from a higher SES background is associated with enhanced gray matter formation and functional connectivity, ultimately leading to more efficient cortical networks in adulthood [43]. 

While much of the research investigating SES-related differences in brain function has been accomplished using rsfMRI, rsEEG has recently gained substantial momentum. One clear advantage of rsEEG is that it is portable, less expensive, and significantly less invasive than MRI, allowing for research in diverse low-income environments. As a result, rsEEG research is more inclusive of international populations and children reared in extreme poverty/adverse conditions than is often observed in MRI research. In rsEEG data, researchers focus on how the overall power or amplitude of specific frequency bands shifts over the course of development. The bands are not universally defined, but are generally categorized as delta (0.5–4 Hz), theta (4–8 Hz), alpha (8–12 Hz), beta (12–30 Hz), and gamma (30–50 or higher Hz) [44]. The typical development trajectory of rsEEG involves an overall decrease in EEG power (average across all frequencies) from late childhood to early adulthood [45,46]. This reduction in EEG power is correlated with a decrease in the volume of gray matter, which occurs during the process of synaptic pruning. During this process, the quantity of synapses that evoke EEG signals diminishes, consequently leading to a decrease in the amplitude of the EEG signal [47,48,49]. 

When examining the developmental trajectories of rsEEG within distinct frequency bands, it is observed that this overall power reduction affects the low frequencies more consistently, with the power of absolute delta and theta frequencies decreasing and the power of alpha and beta frequencies slightly increasing [50,51,52,53,54,55]. The mechanism behind this different effect on the power of low- and high-frequency oscillations during development is relatively understudied [46]. It has been proposed that low frequencies originate from synchronous local neural activity between cortical neurons, and a reduction in synapses involved in this activity could lead to a significant loss of power [48]. In contrast, the beta frequency band, which is associated with asynchronous activity, resulting in lower EEG power, could potentially see an increase in power with the elimination of synapses, if the pruned synapses are activated in conflict with the dominant adjacent synapses. The alpha frequency band is more influenced by structural changes in the thalamus or thalamocortical relays than changes in cortical gray matter. This is supported by other studies, which show that increasing thalamocortical connectivity is associated with increased alpha activity in development [56]. 

Studies of school-aged children from low-SES backgrounds, defined as coming from very low-income households and/or having an illiterate mother, display a different pattern in EEG power distribution, with higher values of overall Absolute Power (AP) relative to children from higher SES backgrounds. This phenomenon has been interpreted as evidence of a maturational lag in brain development [53,57]. Studies that analyzed frequency-specific power show higher delta and theta AP in children from low-SES households or high-risk children in Mexico [58,59]. In higher frequencies, like alpha and gamma, there is an increase in AP at frontal regions for children from higher SES backgrounds compared to children from lower SES backgrounds. Researchers have also identified consistent SES-related differences in higher frequency bands in studies conducted in the US and Europe [60,61,62], with children from lower SES homes exhibiting significantly lower frontal gamma power compared to their higher SES peers [61]. In these studies, income status, maternal education, and maternal occupation are all associated with these reductions in gamma power over the frontal channels [61]. This reduced gamma activity is also observed in children with poorer language outcomes and attention [63,64,65]. Thus, in children from low-SES backgrounds, reduced gamma-band activity over the frontal areas may be an early indicator of a greater subsequent risk for poor language outcomes.

While absolute power is most commonly utilized in rsEEG studies, some argue that relative power (RP—the proportion of frequency-band power compared to total power of all frequency-bands) better addresses issues related to cortical thickness over the course of development [51]. Moreover, although RP findings are closely tied to AP findings, the developmental changes in high-frequency RP are more pronounced than in AP. There are well-established trends in rsEEG RP changes throughout development: there is a reduction in delta and theta RP, and a corresponding increase in alpha and beta RP with advancing age [3,48,58,59,66,67,68,69]. Specifically, children in high-risk situations in Mexico (based on maternal education, more than 4 siblings, and sharing a home with families with similar characteristics) demonstrate differences in lower frequency ranges, including higher delta and theta RP in frontal brain regions, and less alpha RP in posterior brain regions compared to children in more economically advantageous environments [58,59]. These rsEEG differences observed between children in high and low-risk situations diminishes with age, although differences in frontal theta and occipital/left temporal alpha bands persist at 6 years of age [58,59]. 

It is critical to acknowledge that, while these studies demonstrate a link between SES and brain development, it is the larger environmental context associated with SES which shapes brain development. Specifically, factors which are intimately tied to an individual’s SES, such as enhanced exposure to stress and reduced environmental enrichment, are likely the mechanisms which drive brain development and subsequent cognitive outcomes [19,70,71,72]. Across both rsMRI and rsEEG studies, SES is thought to exert its influence on neural development and cognitive outcomes through two pathways which are often correlated. 

The first pathway suggests that mothers with lower educational attainment provide less linguistically diverse vocabulary input to their children in the home environment, in turn affecting the development of the language processing system, including the left inferior frontal gyrus, visual word form area, and perisylvian cortex. Specifically, studies have shown that a lack of diverse vocabulary input negatively impacts child vocabulary development through language-specific neural regions, such as the left inferior frontal gyrus [29]. 

The second pathway is related to lower income, which is thought to result in a more stressful and less structured home environment, due to caregivers’ difficulty accessing resources (food, shelter, transportation, etc.). This in turn affects the developing child’s cognitive control system, including the hippocampus, prefrontal cortex, and amygdala. For example, when children experience higher physiological stress there is a negative impact on working memory, an aspect of cognitive control, through working memory prefrontal neural activity [73,74,75,76]. 

Despite evidence of distinct pathways, maternal education and income are highly correlated across households: individuals with a college degree make nearly 250% more than those with less than a ninth-grade level of education and 130% more than those with a high school diploma [77]. Despite this correlation, few studies have investigated the differential or overlapping effects of each construct on child cognitive development. In one study investigating the effects of both maternal education and income on child development, Khanam and Nghiem (2016) found that both constructs predicted children’s literacy and math scores, however, only income predicted differences in receptive vocabulary and spatial perception (as measured by matrix reasoning) [78]. Other studies have also provided evidence that income and maternal education influence language development [79,80,81]; however, each of these studies emphasizes the mediating role of other environmental factors, such as parenting style, physical environment, health, and stress. Of note, researchers have found independent, non-overlapping effects of each construct, with income having a stable effect over the course of development on children’s later success, while maternal education has more of an influence during infancy [81]. Thus, by concentrating on a single measure of SES, we are oversimplifying existing theoretical models of the relationship between SES and child development as the mechanisms related to SES are largely ignored. In the next sections, we provide more detailed evidence for the overlapping, yet unique influences of maternal education and income on children’s environmental experiences, brain development, and cognitive outcomes. 

### 1.3. Pathway 1. Maternal Education, Language Experiences, and Brain Development

**Figure 1:**
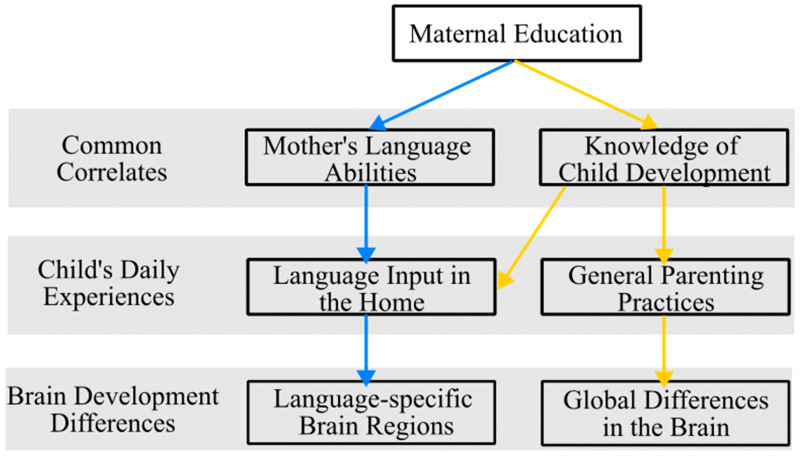
Theoretical model depicting the different pathways by which maternal education influences children’s daily experiences and subsequent brain development. Blue and yellow arrows distinguish different pathways by which maternal education relates to brain development.

A wealth of research has established, that, on average, higher levels of maternal education are related to maternal intellectual quotient (IQ) [82] and the quality of language input in the home [83]. Studies examining maternal IQ and child cognitive outcomes have specified that the home environment predicts child cognitive outcomes, above and beyond maternal IQ, and thus, variability in children’s environments related to the quality of language input is a stronger predictor of child outcomes [84,85]. Specifically, higher maternal education is linked to children hearing more diverse words [86], more complex syntax [87,88,89], and experiencing more conversational turns [29,90,91,92,93,94]; all of which are linked to positive language outcomes. However, the reasons why maternal education influences the quantity and quality of language interactions is less clear [95]. One hypothesis is that higher levels of education results in greater knowledge of child development, including an understanding of the benefits a rich language environment can have on child outcomes (see Figure 1). Supporting this claim, parents’ knowledge about child development mediates the relationship between SES and language input [92]. 

An alternative hypothesis is that higher levels of maternal education results in an increase in lexical richness and grammatical complexity of mothers’ speech (see Figure 1). Supporting this claim, mothers with higher levels of educational attainment talk more, use a richer vocabulary, and produce longer utterances than those with lower educational attainment who had similar beliefs about children’s language abilities [96]. Further, for Spanish–English bilingual children, the effects of maternal education are often only observed in the language in which the mother received her highest level of education, indicating the mother’s experiences in the education system influenced her language abilities in each language, which then influenced how she uses each language with her child [97]. In both cases, maternal education results in a higher quantity and quality of language used by the parent when speaking to the child. In the first argument, these differences in the language environment are due to a better knowledge of child development in general, likely bleeding into other areas of the child’s daily experiences, while in the second, these effects are specific to the language the child hears from the mother. The difference between these arguments is subtle, but may have important differential impacts on brain development.

It appears to be these daily language interactions that lead to differences in the development of language-specific brain regions in children whose mothers have lower levels of educational attainment. Specifically, structural and functional differences in brain development related to SES are due to a deprivation of meaningful language stimulation which causes early proliferation and pruning, resulting in inefficiencies in language outcomes. For example, Noble et al. [5,76] demonstrated that children from lower SES homes have decreased volume in language-specific regions (left inferior frontal gyrus (LIFG) and left superior temporal gyrus (LSTG)), which they attribute to accumulating language deprivation over children’s lifetimes; however, children’s language environments were not directly measured. Expanding this line of work to the examination of brain stiffness and organization, Schneider, McIlvain, and Johnson [98] reported that changes in neural tissue composition are sensitive to malleable aspects of the environment, specifically language input, whereas tissue organization is more strongly associated with vocabulary outcome. Importantly though, these findings were independent of maternal education, indicating that language input, above and beyond maternal education, relates to the structural organization of children’s brains. 

Related to brain function, studies have indicated that there are SES-based differences in the recruitment of neural regions that are necessary for language processing. Raizada et al. [99] reports that child language skills mediate the relationship between SES and functional brain activation in the bilateral IFG. More specifically, Romeo et al. [94] reported that it is conversational turns between caregivers and children that contributes to differences in left IFG activation and subsequent vocabulary development. Similar to Schneider, McIlvain, and Johnson [98], these results were independent of maternal education, indicating that differences in children’s environments are more likely to predict functional development and language outcomes than SES in and of itself.

Maternal education and language input have further been shown to influence the development and maturation of cortical networks [4,60,62,100,101]. Generally speaking, measures of high-quality language input have been associated with lower relative power in low-frequency bands (theta) and higher relative power in high-frequency bands (beta, gamma) in infant EEG studies [100,101]. These same frequencies are also predictive of children’s language outcomes: decreases in lower frequencies (theta) and increases in higher frequencies (alpha, beta, gamma) are positively associated with children’s expressive and receptive vocabulary skills. Despite a clear association between rsEEG, maternal education, and language processes, only a few studies have studied the inter-relationship between these variables. Cantiani et al. [60] found that children from high SES homes had more positive central gamma power at 6 months and a longer mean length of utterance at 24 months of age. These studies provide foundational evidence supporting the inter-relationship between maternal education, rsEEG, and language skills. More recently, Schneider et al. (under review) reported unique pathways by which environmental experiences related to SES influence cognitive and neural development in school-aged children. Specifically, changes in the gamma frequency were linked to both maternal education and vocabulary outcome, but not income. 

While most research examining the link between SES and brain development has focused primarily on the correlations between each construct, recent work has sought to consider pathways by which SES-related differences in brain structure and function emerge. Several recent studies have revealed that adult–child conversational turns—that is, contingent, responsive verbal interactions—mediate links between SES and brain structure [94,102] and function [29] in language-supporting regions. Caregiver self-reports of cognitive stimulation in the home further mediate the link between SES and cortical thickness [103]. These structural and functional MRI studies suggest that cognitive and linguistic stimulation in the home may be the pathway by which socioeconomic differences in brain development emerge and impact language and cognitive outcomes [104]. 

### 1.4. Pathway 2. Income, Stress Related Experiences, and Brain Development

**Figure 2:**
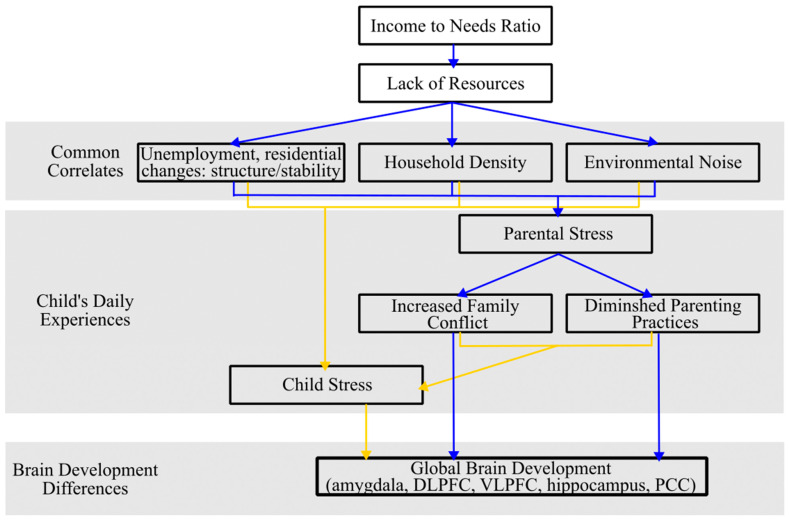
Theoretical model depicting different pathways by which income to needs ratio influences children’s daily experiences and subsequent brain development. Blue and yellow arrows distinguish different pathways by which income relates to brain development.

The income-to-needs (ITN) ratio, a commonly reported proxy for income, is determined by dividing the total income of the household by the poverty threshold, which is adjusted according to the size of the family for the specific year the data is collected [105]. Families with a higher ITN ratio generally possess greater economic resources, which has been shown to be important for child brain development [2,104,106] (see Figure 2). Most notably, increasing low-SES families’ income via a poverty reduction intervention has been associated with greater rsEEG power in the mid-to high-frequency bands [107]. 

The impacts of income are most pronounced during the preschool and early school years, particularly when low income is a persistent condition, and when the level of poverty is severe [108]. Despite the impact of income on brain development, it is crucial to understand that these adverse neural outcomes are not directly caused by income, but rather mediated by limited access to prenatal care, maternal nutrition, perceived stress, and psychological distress, associated with living in a lower income environment [109,110,111,112,113,114,115]. 

Numerous studies have delved into the intricate relationship between income and stress, highlighting the multifaceted nature of this relationship (see Figure 2). A study by Attar et al. [116] revealed that low-income fourth-grade students in the Chicago metropolitan area experienced a 35% increase in stressful life events and challenges within a year compared to their middle-income peers. This is because residing in an economically disadvantaged neighborhood can serve as a persistent stressor, as these communities face higher rates of unemployment and frequent residential changes. These same neighborhoods are also characterized by limited resources and heightened crime rates which cause increased physiological and psychological stress [108,117,118,119]. In addition, families with a lower ITN ratio usually live in a more crowded household and noisier environment, which leads to heightened physiological and psychological stress [120,121,122]. Furthermore, parental emotional distress, often triggered by low income or indebtedness, can result in increased inter-family conflict and the diminished efficacy of parenting practices. Both of these factors have cascading negative effects on children’s behavioral, health, and cognitive outcomes [111,123,124,125].

Evidence derived from empirical research indicates that exposure to stressful events and environments is a significant contributing factor to the observed disparities in brain structure and function among children from lower-income households [106]. Stressors common in low-income families significantly impact the amygdala, dorsolateral, and ventrolateral prefrontal cortex (DLPFC and VLPFC, respectively) [126,127]. These brain regions play a crucial role in regulating emotions, controlling cognition, executing functions, and promoting goal-oriented behaviors [128,129,130]. According to a report by Kim et al. [128], exposure to chronic stressors during childhood serves as a mediator between low-income conditions in childhood and decreased activity in the DLPFC and VLPFC in adults. This suggests that the stressors associated with low-income environments can have long-term effects on brain function. The hippocampus, central to many cognitive functions and emotional processes, also exhibits developmental differences due to stress from low-income households, which explains variations in long-term memory, learning, control of neuroendocrine functions, and the modulation of emotional behavior [131]. It has been reported that lower chronic stress is associated with higher hippocampal volume, which is associated with higher memory performance [108,131,132,133,134]. Furthermore, repeated and prolonged stress leads to the shortening and debranching of dendrites in the hippocampus, a decrease in gray matter volume (GMV) in the anterior cingulate, hippocampus, and parahippocampal gyrus, and induces opposing effects on structural plasticity, such as dendritic atrophy and excitatory synapse loss in the hippocampus and prefrontal cortex [135,136,137]. 

Childhood stress not only affects the brain’s structure but also alters the functional connectivity of neural networks [138]. Philip et al. [139] found that individuals who experienced early life stress showed a greater dissociation between the executive and default mode networks. Specifically, the left dorsolateral prefrontal cortex (DLPFC) exhibited increased local connectivity with the left middle frontal gyrus, while the right DLPFC showed negative connectivity with the inferior parietal lobule compared to the control group. Another rsfMRI study found that higher levels of early life stress are linked to reduced global connectivity and hub-like features in the left DLPFC [138]. Kim et al. [128] reported a positive coupling between the amygdala and the VLPFC in individuals from higher-income households in comparison to low-income households, with greater VLPFC activity resulting in decreased amygdala activation during emotion regulation.

Building upon the comprehensive analysis of rsMRI studies, rsEEG studies have sought to pinpoint the impact of income and stress on brain function. Generally, during development, there is a reduction in delta and theta Relative Power (RP), and a corresponding increase in alpha and beta RP with advancing age [53,58,59,66,68,69]. However, exposure to stressful environments and life events, which are commonly observed in children from low-income households, as previously discussed, has the potential to disrupt this developmental trajectory. Elevated physiological stress levels in mothers, typically gauged by hair cortisol concentration, is correlated with a relative increase in lower frequency power and a relative decrease in higher frequency power in their 6–12-month-old infants [62]. This alteration in the brain’s maturational journey is often considered a sign of possible delay in cognitive development, working memory, learning, and attention [4,62,140,141,142,143]. The same results are observed in other rsEEG studies with older children [4,58,59], where children from low-income homes exhibit poorer performance on language tasks and distinct rsEEG patterns, including increased theta power and decreased alpha power. While these studies independently evaluated income and stress, they consistently yielded similar results, providing additional evidence supporting the assertion that stress functions as a mediator in the relationship between income and brain development. Moreover, among older adults, it has been observed that those who have experienced cumulative life stress exhibit elevations in delta power, which has been associated with the emergence of Alzheimer’s disease and mild cognitive impairment, suggesting that income and stress have long lasting effects on brain development [38]. 

Researchers employing machine learning algorithms in an attempt to diagnose stress are contributing valuable insights into potential rsEEG characteristics that could effectively identify individuals exhibiting symptoms of stress [144,145]. Baumgartl et al. [144] accurately classified individuals with symptoms of high stress at a rate of 81.33% based on significantly higher power in lower frequencies, and significantly lower power in higher frequencies. In the analysis of source-level cortical rsEEG activity related to stress, Vanhollebeke et al. [146] found an increase in alpha power, indicative of decreased cortical activity in both the left and right precuneus and the right PCC, as well as an increase in functional connectivity between the left and right precuneus. This observed decrease in activity within the precuneus/PCC, a crucial component of the default mode network (DMN), may indicate a disruption in an individual’s self-referential neural processes. Income-related, stress-dependent differences in rsEEG also have important implications for children’s cognitive development, as researchers have found that increased rsEEG theta in toddlerhood [147] and during the school years (Schneider et al., under review), are associated with income status, and relates to lower scores on measures of IQ and working memory. 

## 2. Understanding the Role of Cultural Context When Examining SES-Related Differences in Brain and Cognitive Development

**Figure 3:**
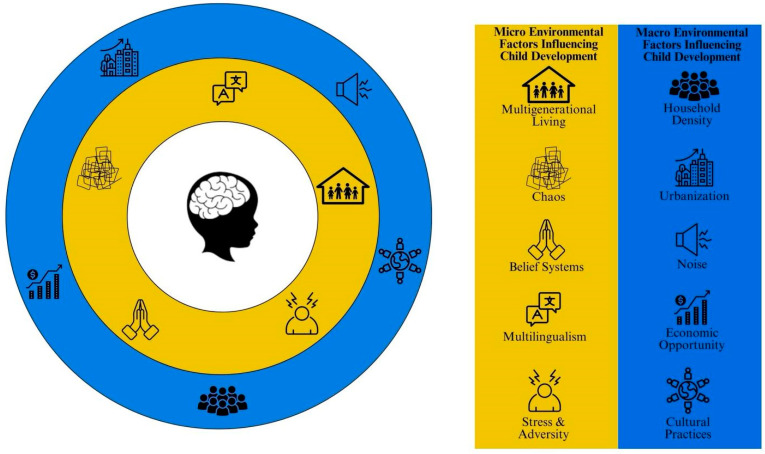
Micro and macro environmental factors that differentially influence child cognitive and brain development. Items highlighted in yellow represent micro-environmental factors that are more proximal to the child’s development, while items highlighted in blue represent macro-environmental factors that are more distal to the child’s development.

To date, much of the research on SES-related differences in brain development draws conclusions from comparisons of two groups that differ markedly in one or more respects (e.g., SES, race, ethnicity, first language, bilingualism), [148]. This is problematic as important mechanisms through which SES might act on the developing brain to influence cognitive outcomes are often overlooked. Despite a number of recent studies demonstrating the mediating role of language input and physiological stress on brain and language development, there are a number of other important confounds which are related to SES that should be considered (see Figure 3). For example, culture, multilingualism, and household density may influence brain and cognitive development in unique ways that have not yet been examined [148]. Here, we outline confounds of SES that should be considered in future studies of SES and brain development. Consideration of these factors in future studies of the relationship between SES and brain development would enhance the scope of applicability and facilitate more informed policymaking to positively impact a broader demographic. 

### 2.1. Consideration of Multigenerational Households

The 30-million-word gap has been a topic of debate in child development research since Hart and Risley first published their landmark study in 1995 [83]. They famously reported a difference in language exposure between children of different socioeconomic status (SES) homes by collecting and analyzing home speech samples of low-SES and high SES families. Specifically, they found that, by the age of 4, children from higher SES families heard, on average, 30 million more words than children from lower SES families [83]. Given that research has shown that the amount of language children hear impacts both brain structure and function [29,94,102], there has been an ever-growing interest in studying the home language environment. 

A critical issue with existing studies relating children’s language environments to brain development is that they often fail to consider differences in cultural beliefs about multi-generational cohabitation. Households which include grandparents and/or extended family are common in collectivist societies throughout South America, Africa, and Asia [149], and are increasing in prevalence among developing countries [150]. Due to cultural and economic shifts, the number of multiple generational homes in the US has quadrupled in the last 50 years [151]. How this might influence the child’s language development is still an area of debate, as multigenerational households can offer multiple communication partners but lead to reductions in one-on-one interactions. Research that expands the definition of language exposure to include both language input from a primary caregiver and language produced to and around the child by others (e.g., siblings, aunts/uncles, other primary caregivers, etc.) results in a vastly diminished word gap on the basis of SES while also taking into account socio-cultural differences in family structure [152,153,154]. Specifically, when considering all speech in a child’s environment instead of just maternal or parental speech, children from different SES groups do not hear vastly different language quantities [152,153]. This is critical to consider in future studies of language input and brain development, as lower-SES families and racial and ethnic minority groups, including foreign-born, Asian, Black, and Hispanic Americans are more likely to live in larger, multigenerational households (also termed co-residence) [151,155]. Thus, children commonly examined in studies of SES-related differences in brain and cognitive development are more likely to experience environments with numerous sources of language input. The failure to consider how these variable sources of language input mediate the relationship between SES and brain development, therefore, severely impedes our ability to extrapolate a holistic perspective of child brain development. 

### 2.2. Consideration of Caregiver Practices and Belief Systems

Given the importance of language input in brain development, recent research has examined how modifying the home language environment can promote brain development in children from lower SES households. Romeo et al. [156] revealed a positive link between improved turn-taking (following intervention) and cortical thickening in the left inferior frontal and supramarginal gyri. Notably, the supramarginal gyrus mediated the relationship between turn-taking changes and children’s language development following intervention. 

Despite the positive effects of these interventions, an unaddressed issue with much of this research is the lack of consideration of caregiver beliefs and practices about language input in the home environment. There is growing evidence of SES and race-based differences in parents’ knowledge about general infant development and early language and literacy development [157,158,159]. For example, despite agreement between mid SES Black mothers and White mothers on a number of caregiving practices, low-SES Black mothers believe that their children’s language develops naturally and does not require child-directed speech input [160]. Furthermore, Luo et al. [161] demonstrated that bilingual, Hispanic parents with higher levels of education and income were more likely than their lower SES counterparts to recognize the benefits of supporting language development in the home and school contexts. Although these studies focus primarily on caregiver beliefs and practices related to language input, their relevance for brain development should not be overlooked, as language input is consistently highlighted in studies of SES-related differences of brain development.

### 2.3. Consideration of Underrepresented Cultures

In an excellent review of the state of child development research, Draper et al. [162] highlight the lack of global representation in the field between 2006–2010. Fewer than 3% of participants in studies of child development were from Central and South America, Africa, Asia, or the Middle East, despite these contexts constituting 85% of the world’s population [163]. This lack of representation is a shortcoming in existing research, as the environmental experiences of children from low-SES environments in the United States may differ drastically from those of children in non-WEIRD cultures. For example, although the influence of SES on child development is stable across countries [164], a meta-analysis of 39 low-income and middle-income countries found that the association between these variables systematically differs based on unique environmental experiences, such as country-level adult illiteracy, infant mortality, and food insecurity [165]. Similarly, in China, maternal education is the strongest predictor of children’s educational attainment, however, this relationship is fully mediated by parenting practices and children’s learning attitudes, which differ from Westernized parenting practices and child attitudes [166]. This lack of global representation severely limits our understanding of the role culture plays in brain and cognitive development.

As previously stated, language input mediates the pathway between SES and brain development [94], however, the exposure to language input substantially differs not just across households, but across cultures [167]. In Tseltal Mayan and Papua New Guinea cultures, children are rarely directly addressed by caregivers, but experience higher rates of overheard speech or speech that is not directed to them [168]. Similarly, children in rural farming communities hear significantly less input from caregivers than children in urban regions, where the bulk of extant research studies are conducted [169]. Importantly though, children in these global communities manage to extract the linguistic information they need to learn new words despite minimal directed speech [168]. Therefore, while westernized standards promote child directed speech, something commonly targeted in interventions aimed at improving children’s language environments, a number of studies in other regions of the globe indicate that overheard speech is equally as beneficial for the development of children’s language skills [170,171], and likely brain development. 

However, to date, there are no studies examining how overheard speech mediates the relationship between SES and child brain development. Given that rsEEG is relatively low in cost and mobile, it offers an opportunity to explore how higher levels of overheard speech relate to variability in brain development across a range of cultures. 

### 2.4. Consideration of Bilingual Populations

There are more bilingual and multilingual children around the world who grow up learning and using two or more languages than there are monolingual children [172,173]. Reported rates of bilingualism in places such as Europe (67%), Canada (55%), India (25%), and the United States (20%) indicate that bilingualism is both prevalent and growing [174]. Rates of bilingualism in the United States are even higher among children (26% of 5–17-year-olds nationwide), with certain regions reporting nearly 50% of the child population being bilingual (44% in California [175]) and 49% in Texas [176]. Despite the dominance of bi- and multi-lingualism in global and local contexts, the influence of bilingualism on child brain development remains poorly understood in relation to what is known about monolingual populations [172]. In fact, many studies of brain and language development in the United States have historically excluded bilingual participants. 

Monolingual and bilingual children experience different language environments, which, in turn, may result in changes in the development of cortical networks underlying language and cognitive development. Unlike monolingual families, bilingual families differ in which languages are spoken by whom, and specifically when each language is spoken to the child [177]. For example, in a study of Spanish–English bilingual mothers, the effect of maternal education on child language abilities was only observed in the language in which the mother received her highest level of education [97]. Additionally, research has shown that language input from older siblings, but not from parents, is associated with stronger lexical, grammatical, and narrative abilities in English among young bilingual speakers [178,179]. The language environment of bilingual children has been shown to be highly dynamic as different individuals join or leave the household [180]. These findings are important as they highlight variability in children’s language environments that are unaccounted for when conducting research in monolingual populations only. 

It is especially important to consider variability in bilingual children’s environments, as research has shown that bilingualism can serve as an important neuromodulator [181,182,183]. Specifically, variability in bilingual language experience modulates neural adaptability during development [181,183,184], finding that bilingualism permanently shifts underlying brain mechanisms, leading to long-term structural and functional changes [185,186,187,188,189]. This suggests that life experiences related to bilingualism can ultimately induce brain changes, such as volumetric changes, differences in spatial distribution and functional connectivity, as well as more distributed cortical activation, which converges in frontal regions [182,185,187,188,190]. Patterns of structural differences appear to depend on whether two languages were acquired simultaneously from birth, or sequentially before age five [191], suggesting that timing of bilingualism interacts with brain development [192]. Thus, bilingual children’s early language experiences not only differ from monolingual children and result in different trajectories for brain development, but the age at which they experience bilingual language input results in variability in neural circuitry as well. Despite these associations, little to no research has directly measured the quantity and quality of language input by bilingual caregivers within each language, and how these differences influence the trajectory of children’s brain development, limiting our ability to generalize existing findings to the increasingly dominant bilingual population.

### 2.5. Current Demographic Shifts: Household Density, Urbanization, Chaos, and Noise

Stress is also a key mechanism linking children’s exposure to poverty-related adversity and brain development. Chronic stressors related to urbanization include higher household density, noise, and household chaos, which alter the physiologic response to stress, leading to potentially teratogenic effects of stress-related hormones on the developing brain and to a range of adverse outcomes in the development of executive function, socio-emotional, language, and the brain [193,194,195,196,197,198,199,200,201]. Understanding how the factors associated with urbanization influence caregiver and child stress and, in turn, impacting child brain development, is critical as the world is currently experiencing a wave of urbanization and population growth. Individuals living in more urban regions (based on measures of population density and duration of residency) exhibit differences in brain volume, cortical surface area, and brain network connectivity in the medial prefrontal cortex and cerebellum, related to depressive symptoms and difficulties perspective-taking. Importantly, the effects of living in a more urban area on brain development are the greatest in the cerebellum during childhood, and in the prefrontal cortex from childhood to adolescence [202], suggesting that urban environments alter different aspects of the brain across the course of development. Understanding how chronic stressors associated with urbanization, such as higher household density, noise, and household chaos, influence brain and language development is vital for addressing differences in these domains on the basis of SES.

Household density is often measured as the number of people in a household divided by number of rooms (or bedrooms) and is more common among lower income households, with the available number of rooms per household member being significantly lower in lower income neighborhoods [122,203]. Household density is often viewed negatively, with past research indicating that household density is negatively correlated with language outcomes in children [204,205,206,207]. However, this deficit-based perspective regarding the relationship between household density and child language development lacks cultural responsivity. One key issue is that most studies of household density fail to differentiate adults from children in the household. For example, when using traditional ratios of number of people to number of bedrooms in the home, household density is negatively associated with language outcomes. Importantly though, when the family make up is calculated as adult to child ratio, there are positive outcomes for children having more adults in the home [122]. Furthermore, Sperry, Sperry, and Miller [152] revealed that when speech overheard by children from other adults in the home was included in calculations of the children’s language input, there was no significant relationship between vocabulary and SES. Similar work related to brain development has not yet been conducted, but taking a more nuanced approach to household density in the future may provide greater insights into how individual members of the household might support or detract from a child’s language environment. 

Another stressor related to urbanization is the presence of noise in both the child’s home and community environment. Noise can mask the speech signal of interest, making it difficult for children to access meaningful language input. This is particularly concerning as research has shown that children raised in noisy households face an increased risk of experiencing language, reading, and academic difficulties [208,209,210], as well as deficits in cognitive abilities that underlie language processing, such as working memory and attention [211,212]. Noisy environments are more common in high density households and can pose a risk to children’s cognitive and brain development. Simon et al. [213] reported that children exposed to excessive levels of noise exhibited reduced cortical thickness in the left IFG, a region that is crucial for language processing. Furthermore, a study of 8–12-year-olds exposed to school road-traffic noise reported enhanced connectivity in the subcortical auditory pathway, suggesting that prolonged noise exposure may accelerate the neural maturation of the auditory pathway [214]. These findings add to a growing literature that explores the extent to which environmental noise is associated with neurobiological development underlying language and cognitive outcomes in children. Despite evidence of a negative association between noise and child brain and language development, children exposed to intense environmental noise may initially show an *increased* ability to tune out auditory distractors [215], making them better at learning from the language input they are provided. Thus, while noise in the environment is thought to typically have a negative effect on children’s brain, vocabulary, and reading development, it is possible that noise exposure may initially offer an advantage to young children. Given that reducing noise in high density households may be difficult, it would be beneficial for future research to take a strengths-based approach to understanding which aspects of environmental noise may promote child brain and language development. 

Lastly, urbanization may result in increased levels of chaos, a common measure of chronic stress, however, the measures utilized to assess chaos in the home may also be susceptible to examiner bias. Chaos is most commonly measured based on caregiver self-report using the Confusion, Hubbub and Order Scale (CHAOS) or by examiner observation using the Home Observation for Measurement (HOME) of the Environment. These two measures capture unique and different aspects of the environment that should be considered in context. The same environment can be viewed as chaotic to one person and a warm, active, lively household to another. In a study examining the psychometric properties of the HOME, there was substantial variability in its ecological validity across diverse cultural contexts [216]. Similarly, the CHAOS includes items with language that may be stigmatizing and off-putting to families from diverse backgrounds (e.g., “it’s a real zoo in our house”, “there is often a fuss going on at our home”) [217]. Making assumptions without considering the larger context which influences behavior may reinforce stigmatization among communities with different cultural values or life experiences [217].

Urbanization may result in individuals being exposed to multiple stressors simultaneously, making it challenging to quantify the impact of a specific environmental factor on child brain development [218]. In summary, the multifaceted impact of urbanization related to household density, environmental noise, and chaos can influence language, cognitive, and brain development through various pathways, including overcrowding and caregiver stress. Recognizing and understanding these intricate relationships is essential to develop effective strategies to support language development in low-SES household settings.

### 2.6. Considerations for Texture and Style of Hair in Neuroimaging Techniques

One factor that may substantially influence the generalizability of rsEEG research is rooted in the technique by which this data is collected. EEG data collection requires access to the hair and scalp, indicating that the practical usage of EEG is affected by physical factors such as the texture and style of hair, as well as sociocultural factors relevant to having one’s hair touched by someone else [219]. These physical factors have historically, and currently, resulted in the exclusion of groups of people, particularly Black communities [220,221,222,223,224,225,226]. As such, most EEG research studies have been dominated by individuals with privileged identities, particularly White, college-educated people [222,223,227,228]. However, due to long-standing systems of racial oppression, the representation of samples is often not reported, and findings are generalized as if they were drawn from representative samples [229]. These same systems of racial oppression and structural racism have resulted in a higher distribution of Black families living in lower-income environments [230,231], indicating that rsEEG studies examining socioeconomic differences may not be representative of individuals from diverse racial groups. To promote diversity in the participants recruited for EEG studies, researchers should consider engaging Black researchers in both the development and execution of EEG research, as this has been shown to result in a more positive experience for the participant while also collecting useful data [232,233,234,235]. Providing hair care products that are conducive to Black hair may also promote inclusivity and make the cleanup process less burdensome for participants [233,236]. Although new organizations such as Black in Neuro (blackinneuro.com) and SPARK Society (sparksociety.org) are conducting foundational work to raise awareness about systemic racism in neuroscience, researchers can also consider ways by which their EEG methods can be revised to promote diversity, equity, and inclusion.

## 3. Conclusions

SES is known to influence brain development through accumulated environmental experiences. Specifically, there appear to be two dominant pathways which account for this association: (1) maternal education results in differences in the quality and quantity of language input children hear, having downstream effects on the development of language-related brain regions and (2) differences in income alter physiological stress in the home, in turn impacting regions of the brain that are critical for self-regulation and working memory. While relatively underutilized in studies of neuroimaging, we provide evidence that rsEEG can uncover important differences in brain function that are subserved by these socioeconomic differences in environmental experiences. Furthermore, it is a cost effective and mobile technology that can capture brain development across a variety of households and cultures. This is critical in providing a more comprehensive understanding of the relationship between SES and brain development, one that includes the broader cultural context related to factors which may interact with SES or exert unique influences on brain development that are often overlooked, such bilingualism, household density, parenting practices, environmental noise, and chaos. Considering the broader cultural context in SES-related studies of brain development will advance the field of developmental cognitive neuroscience and provide researchers with a more generalizable, holistic model of child brain development. 

## References

[1] Farah M.J. (2017). The Neuroscience of Socioeconomic Status: Correlates, Causes, and Consequences. Neuron.

[2] Duncan G.J., Magnuson K. (2012). Socioeconomic Status and Cognitive Functioning: Moving from Correlation to Causation. WIREs Cogn. Sci..

[3] Harmony T., Alvarez A., Pascual R., Ramos A., Marosi E., Díaz De León A.E., Valdés P., Becker J. (1988). EEG Maturation on Children with Different Economic and Psychosocial Characteristics. Int. J. Neurosci..

[4] Maguire M.J., Schneider J.M. (2019). Socioeconomic Status Related Differences in Resting State EEG Activity Correspond to Differences in Vocabulary and Working Memory in Grade School. Brain Cogn..

[5] Noble K.G., Houston S.M., Kan E., Sowell E.R. (2012). Neural Correlates of Socioeconomic Status in the Developing Human Brain. Dev. Sci..

[6] Noble K.G., Houston S.M., Brito N.H., Bartsch H., Kan E., Kuperman J.M., Akshoomoff N., Amaral D.G., Bloss C.S., Libiger O. (2015). Family Income, Parental Education and Brain Structure in Children and Adolescents. Nat. Neurosci..

[7] Olson L., Chen B., Fishman I. (2021). Neural Correlates of Socioeconomic Status in Early Childhood: A Systematic Review of the Literature. Child Neuropsychol..

[8] Otero G.A. (1997). Poverty, Cultural Disadvantage and Brain Development: A Study of Pre-School Children in Mexico. Electroencephalogr. Clin. Neurophysiol..

[9] Duncan G.J., Yeung W.J., Brooks-Gunn J., Smith J.R. (1998). How Much Does Childhood Poverty Affect the Life Chances of Children?. Am. Sociol. Rev..

[10] Cohen S., Janicki-Deverts D., Chen E., Matthews K.A. (2010). Childhood Socioeconomic Status and Adult Health. Ann. N. Y. Acad. Sci..

[11] Evans G.W., Cassells R.C. (2014). Childhood Poverty, Cumulative Risk Exposure, and Mental Health in Emerging Adults. Clin. Psychol. Sci..

[12] Schnack H.G., van Haren N.E.M., Brouwer R.M., Evans A., Durston S., Boomsma D.I., Kahn R.S., Hulshoff Pol H.E. (2015). Changes in Thickness and Surface Area of the Human Cortex and Their Relationship with Intelligence. Cereb. Cortex.

[13] Sowell E.R., Peterson B.S., Thompson P.M., Welcome S.E., Henkenius A.L., Toga A.W. (2003). Mapping Cortical Change across the Human Life Span. Nat. Neurosci..

[14] Tamnes C.K., Herting M.M., Goddings A.-L., Meuwese R., Blakemore S.-J., Dahl R.E., Güroğlu B., Raznahan A., Sowell E.R., Crone E.A. (2017). Development of the Cerebral Cortex across Adolescence: A Multisample Study of Inter-Related Longitudinal Changes in Cortical Volume, Surface Area, and Thickness. J. Neurosci..

[15] Brouwer R.M., Klein M., Grasby K.L., Schnack H.G., Jahanshad N., Teeuw J., Thomopoulos S.I., Sprooten E., Franz C.E., Gogtay N. (2022). Genetic Variants Associated with Longitudinal Changes in Brain Structure across the Lifespan. Nat. Neurosci..

[16] Bethlehem R.A.I., Seidlitz J., White S.R., Vogel J.W., Anderson K.M., Adamson C., Adler S., Alexopoulos G.S., Anagnostou E., Areces-Gonzalez A. (2022). Brain Charts for the Human Lifespan. Nature.

[17] Grady C. (2012). The Cognitive Neuroscience of Ageing. Nat. Rev. Neurosci..

[18] Luna B., Bauer P. (2009). Developmental Changes in Cognitive Control through Adolescence. Advances in Child Development and Behavior.

[19] Hackman D.A., Farah M.J. (2009). Socioeconomic Status and the Developing Brain. Trends Cogn. Sci..

[20] Mackey A.P., Finn A.S., Leonard J.A., Jacoby-Senghor D.S., West M.R., Gabrieli C.F.O., Gabrieli J.D.E. (2015). Neuroanatomical Correlates of the Income-Achievement Gap. Psychol. Sci..

[21] Hanson J.L., Hair N., Shen D.G., Shi F., Gilmore J.H., Wolfe B.L., Pollak S.D. (2013). Family Poverty Affects the Rate of Human Infant Brain Growth. PLoS ONE.

[22] Jednoróg K., Altarelli I., Monzalvo K., Fluss J., Dubois J., Billard C., Dehaene-Lambertz G., Ramus F. (2012). The Influence of Socioeconomic Status on Children’s Brain Structure. PLoS ONE.

[23] Lawson G.M., Duda J.T., Avants B.B., Wu J., Farah M.J. (2013). Associations between Children’s Socioeconomic Status and Prefrontal Cortical Thickness. Dev. Sci..

[24] Kim D.-J., Davis E.P., Sandman C.A., Glynn L., Sporns O., O’Donnell B.F., Hetrick W.P. (2019). Childhood Poverty and the Organization of Structural Brain Connectome. NeuroImage.

[25] Moriguchi Y., Shinohara I. (2019). Socioeconomic Disparity in Prefrontal Development during Early Childhood. Sci. Rep..

[26] Rakesh D., Whittle S. (2021). Socioeconomic Status and the Developing Brain—A Systematic Review of Neuroimaging Findings in Youth. Neurosci. Biobehav. Rev..

[27] Sheridan M.A., Sarsour K., Jutte D., D’Esposito M., Boyce W.T. (2012). The Impact of Social Disparity on Prefrontal Function in Childhood. PLoS ONE.

[28] Su M., Li P., Zhou W., Shu H. (2021). Effects of Socioeconomic Status in Predicting Reading Outcomes for Children: The Mediation of Spoken Language Network. Brain Cogn..

[29] Romeo R.R., Leonard J.A., Robinson S.T., West M.R., Mackey A.P., Rowe M.L., Gabrieli J.D.E. (2018). Beyond the 30-Million-Word Gap: Children’s Conversational Exposure Is Associated with Language-Related Brain Function. Psychol. Sci..

[30] King L.S., Camacho M.C., Montez D.F., Humphreys K.L., Gotlib I.H. (2021). Naturalistic Language Input Is Associated with Resting-State Functional Connectivity in Infancy. J. Neurosci..

[31] McDermott C.L., Seidlitz J., Nadig A., Liu S., Clasen L.S., Blumenthal J.D., Reardon P.K., Lalonde F., Greenstein D., Patel R. (2019). Longitudinally Mapping Childhood Socioeconomic Status Associations with Cortical and Subcortical Morphology. J. Neurosci..

[32] Fox M.D., Raichle M.E. (2007). Spontaneous Fluctuations in Brain Activity Observed with Functional Magnetic Resonance Imaging. Nat. Rev. Neurosci..

[33] De Luca M., Beckmann C.F., De Stefano N., Matthews P.M., Smith S.M. (2006). fMRI Resting State Networks Define Distinct Modes of Long-Distance Interactions in the Human Brain. NeuroImage.

[34] Damoiseaux J.S., Rombouts S.A.R.B., Barkhof F., Scheltens P., Stam C.J., Smith S.M., Beckmann C.F. (2006). Consistent Resting-State Networks across Healthy Subjects. Proc. Natl. Acad. Sci. USA.

[35] Mantini D., Perrucci M.G., Del Gratta C., Romani G.L., Corbetta M. (2007). Electrophysiological Signatures of Resting State Networks in the Human Brain. Proc. Natl. Acad. Sci. USA.

[36] Leopold D.A., Murayama Y., Logothetis N.K. (2003). Very Slow Activity Fluctuations in Monkey Visual Cortex: Implications for Functional Brain Imaging. Cereb. Cortex.

[37] Bice K., Yamasaki B.L., Prat C.S. (2020). Bilingual Language Experience Shapes Resting-State Brain Rhythms. Neurobiol. Lang..

[38] Marshall A.C., Cooper N.R. (2017). The Association between High Levels of Cumulative Life Stress and Aberrant Resting State EEG Dynamics in Old Age. Biol. Psychol..

[39] Gellci K., Marusak H.A., Peters C., Elrahal F., Iadipaolo A.S., Rabinak C.A. (2019). Community and Household-Level Socioeconomic Disadvantage and Functional Organization of the Salience and Emotion Network in Children and Adolescents. NeuroImage.

[40] Gao W., Alcauter S., Elton A., Hernandez-Castillo C.R., Smith J.K., Ramirez J., Lin W. (2015). Functional Network Development During the First Year: Relative Sequence and Socioeconomic Correlations. Cereb. Cortex.

[41] Barch D., Pagliaccio D., Belden A., Harms M.P., Gaffrey M., Sylvester C.M., Tillman R., Luby J. (2016). Effect of Hippocampal and Amygdala Connectivity on the Relationship between Preschool Poverty and School-Age Depression. Am. J. Psychiatry.

[42] Tooley U.A., Mackey A.P., Ciric R., Ruparel K., Moore T.M., Gur R.C., Gur R.E., Satterthwaite T.D., Bassett D.S. (2020). Associations between Neighborhood SES and Functional Brain Network Development. Cereb. Cortex.

[43] Tooley U.A., Bassett D.S., Mackey A.P. (2021). Environmental Influences on the Pace of Brain Development. Nat. Rev. Neurosci..

[44] Buzsáki G. (2006). Rhythms of the Brain.

[45] Dustman R.E., Shearer D.E., Emmerson R.Y. (1999). Life-Span Changes in EEG Spectral Amplitude, Amplitude Variability and Mean Frequency. Clin. Neurophysiol..

[46] Lüchinger R., Michels L., Martin E., Brandeis D. (2012). Brain State Regulation during Normal Development: Intrinsic Activity Fluctuations in Simultaneous EEG–fMRI. NeuroImage.

[47] Sakai J. (2022). Functional Near-Infrared Spectroscopy Reveals Brain Activity on the Move. Proc. Natl. Acad. Sci. USA.

[48] Whitford T.J., Rennie C.J., Grieve S.M., Clark C.R., Gordon E., Williams L.M. (2007). Brain Maturation in Adolescence: Concurrent Changes in Neuroanatomy and Neurophysiology. Hum. Brain Mapp..

[49] Lüchinger R., Michels L., Martin E., Brandeis D. (2011). EEG–BOLD Correlations during (Post-)Adolescent Brain Maturation. NeuroImage.

[50] Benninger C., Matthis P., Scheffner D. (1984). EEG Development of Healthy Boys and Girls. Results of a Longitudinal Study. Electroencephalogr. Clin. Neurophysiol..

[51] Clarke A.R., Barry R.J., McCarthy R., Selikowitz M. (2001). Age and Sex Effects in the EEG: Development of the Normal Child. Clin. Neurophysiol..

[52] Cragg L., Kovacevic N., McIntosh A.R., Poulsen C., Martinu K., Leonard G., Paus T. (2011). Maturation of EEG Power Spectra in Early Adolescence: A Longitudinal Study. Dev. Sci..

[53] Harmony T., Marosi E., Díaz de León A.E., Becker J., Fernández T. (1990). Effect of Sex, Psychosocial Disadvantages and Biological Risk Factors on EEG Maturation. Electroencephalogr. Clin. Neurophysiol..

[54] Matthis P., Scheffner D., Benninger C., Lipinski C., Stolzis L. (1980). Changes in the Background Activity of the Electroencephalogram According to Age. Electroencephalogr. Clin. Neurophysiol..

[55] Somsen R.J.M., van’t Klooster B.J., van der Molen M.W., van Leeuwen H.M.P., Licht R. (1997). Growth Spurts in Brain Maturation during Middle Childhood as Indexed by EEG Power Spectra. Biol. Psychol..

[56] Tröndle M., Popov T., Dziemian S., Langer N. (2022). Decomposing the Role of Alpha Oscillations during Brain Maturation. eLife.

[57] Wilkinson C.L., Pierce L.J., Sideridis G., Wade M., Nelson C.A. (2023). Associations between EEG Trajectories, Family Income, and Cognitive Abilities over the First Two Years of Life. Dev. Cogn. Neurosci..

[58] Otero G.A. (1994). Eeg Spectral Analysis in Children with Sociocultural Handicaps. Int. J. Neurosci..

[59] Otero G.A., Pliego-Rivero F.B., Fernández T., Ricardo J. (2003). EEG Development in Children with Sociocultural Disadvantages: A Follow-up Study. Clin. Neurophysiol..

[60] Cantiani C., Piazza C., Mornati G., Molteni M., Riva V. (2019). Oscillatory Gamma Activity Mediates the Pathway from Socioeconomic Status to Language Acquisition in Infancy. Infant Behav. Dev..

[61] Tomalski P., Moore D.G., Ribeiro H., Axelsson E.L., Murphy E., Karmiloff-Smith A., Johnson M.H., Kushnerenko E. (2013). Socioeconomic Status and Functional Brain Development—Associations in Early Infancy. Dev. Sci..

[62] Troller-Renfree S.V., Brito N.H., Desai P.M., Leon-Santos A.G., Wiltshire C.A., Motton S.N., Meyer J.S., Isler J., Fifer W.P., Noble K.G. (2020). Infants of Mothers with Higher Physiological Stress Show Alterations in Brain Function. Dev. Sci..

[63] Barry R.J., Clarke A.R., Hajos M., McCarthy R., Selikowitz M., Dupuy F.E. (2010). Resting-State EEG Gamma Activity in Children with Attention-Deficit/Hyperactivity Disorder. Clin. Neurophysiol..

[64] Behboudi M.H., Castro S., Chalamalasetty P., Maguire M.J. (2023). Development of Gamma Oscillation during Sentence Processing in Early Adolescence: Insights into the Maturation of Semantic Processing. Brain Sci..

[65] Benasich A.A., Gou Z., Choudhury N., Harris K.D. (2008). Early Cognitive and Language Skills Are Linked to Resting Frontal Gamma Power across the First 3 Years. Behav. Brain Res..

[66] Anderson A.J., Perone S. (2018). Developmental Change in the Resting State Electroencephalogram: Insights into Cognition and the Brain. Brain Cogn..

[67] Brito N.H., Fifer W.P., Myers M.M., Elliott A.J., Noble K.G. (2016). Associations among Family Socioeconomic Status, EEG Power at Birth, and Cognitive Skills during Infancy. Dev. Cogn. Neurosci..

[68] Marshall P.J., Fox N.A., Group B.C. (2004). A Comparison of the Electroencephalogram between Institutionalized and Community Children in Romania. J. Cogn. Neurosci..

[69] Uhlhaas P.J., Singer W. (2010). Abnormal Neural Oscillations and Synchrony in Schizophrenia. Nat. Rev. Neurosci..

[70] Callaghan B.L., Tottenham N. (2016). The Stress Acceleration Hypothesis: Effects of Early-Life Adversity on Emotion Circuits and Behavior. Curr. Opin. Behav. Sci..

[71] Leonard J.A., Romeo R.R., Park A.T., Takada M.E., Robinson S.T., Grotzinger H., Last B.S., Finn A.S., Gabrieli J.D.E., Mackey A.P. (2019). Associations between Cortical Thickness and Reasoning Differ by Socioeconomic Status in Development. Dev. Cogn. Neurosci..

[72] Park A.T., Leonard J.A., Saxler P.K., Cyr A.B., Gabrieli J.D.E., Mackey A.P. (2018). Amygdala–Medial Prefrontal Cortex Connectivity Relates to Stress and Mental Health in Early Childhood. Soc. Cogn. Affect. Neurosci..

[73] Qin S., Hermans E.J., van Marle H.J.F., Luo J., Fernández G. (2009). Acute Psychological Stress Reduces Working Memory-Related Activity in the Dorsolateral Prefrontal Cortex. Biol. Psychiatry.

[74] Noble K.G., Norman M.F., Farah M.J. (2005). Neurocognitive Correlates of Socioeconomic Status in Kindergarten Children. Dev. Sci..

[75] Farah M.J., Shera D.M., Savage J.H., Betancourt L., Giannetta J.M., Brodsky N.L., Malmud E.K., Hurt H. (2006). Childhood Poverty: Specific Associations with Neurocognitive Development. Brain Res..

[76] Noble K.G., McCandliss B.D., Farah M.J. (2007). Socioeconomic Gradients Predict Individual Differences in Neurocognitive Abilities. Dev. Sci..

[77] U.S. Bureau of Labor Statistics and U.S. Census Bureau (2023) HINC-01. Selected Characteristics of Households by Total Money Income. Current Population Survey (CPS) Annual Social and Economic (ASEC) Supplement. https://www.census.gov/data/tables/time-series/demo/income-poverty/cps-hinc/hinc-01.html.

[78] Khanam R., Nghiem S. (2016). Family Income and Child Cognitive and Noncognitive Development in Australia: Does Money Matter?. Demography.

[79] Mistry R., Biesanz J., Taylor L., Burchinal M., Cox M. (2004). Family income and its relation to preschool children’s adjustment for families in the NICHD Study of Early Child Care. Dev. Psychol..

[80] Guo G., Harris K.M. (2000). The mechanisms mediating the effects of poverty on children’s intellectual development. Demography.

[81] Erola J., Jalonen S., Lehti H. (2016). Parental education, class and income over early life course and children’s achievement. Res. Soc. Stratif. Mobil..

[82] Lean R.E., Paul R.A., Smyser C.D., Rogers C.E. (2018). Maternal intelligence quotient (IQ) predicts IQ and language in very preterm children at age 5 years. J. Child Psychol. Psychiatry.

[83] Hart B., Risley T.R., Kirby J.R. (1997). Meaningful Differences in the Everyday Experience of Young American Children. Can. J. Educ..

[84] Bradley R.H., Whiteside L., Caldwell B.M., Casey P.H., Kelleher K., Pope S., Swanson M., Barrett K., Cross D. (1993). Maternal IQ, the home environment, and child IQ in low birthweight, premature children. Int. J. Behav. Dev..

[85] Ronfani L., Vecchi Brumatti L., Mariuz M., Tognin V., Bin M., Ferluga V., Knowles A., Montico M., Barbone F. (2015). The complex interaction between home environment, socioeconomic status, maternal IQ and early child neurocognitive development: A multivariate analysis of data collected in a newborn cohort study. PLoS ONE.

[86] Rowe M.L. (2012). A Longitudinal Investigation of the Role of Quantity and Quality of Child-Directed Speech in Vocabulary Development. Child Dev..

[87] Huttenlocher J., Vasilyeva M., Cymerman E., Levine S. (2002). Language Input and Child Syntax. Cogn. Psychol..

[88] Huttenlocher J., Vasilyeva M., Waterfall H.R., Vevea J.L., Hedges L.V. (2007). The Varieties of Speech to Young Children. Dev. Psychol..

[89] Huttenlocher J., Waterfall H., Vasilyeva M., Vevea J., Hedges L.V. (2010). Sources of Variability in Children’s Language Growth. Cogn. Psychol..

[90] Ferjan Ramírez N., Lytle S.R., Kuhl P.K. (2020). Parent Coaching Increases Conversational Turns and Advances Infant Language Development. Proc. Natl. Acad. Sci. USA.

[91] Hirsh-Pasek K., Adamson L.B., Bakeman R., Owen M.T., Golinkoff R.M., Pace A., Yust P.K.S., Suma K. (2015). The Contribution of Early Communication Quality to Low-Income Children’s Language Success. Psychol. Sci..

[92] Rowe M.L. (2008). Child-Directed Speech: Relation to Socioeconomic Status, Knowledge of Child Development and Child Vocabulary Skill. J. Child Lang..

[93] Schwab J.F., Lew-Williams C. (2016). Language Learning, Socioeconomic Status, and Child-Directed Speech. WIREs Cogn. Sci..

[94] Romeo R.R., Segaran J., Leonard J.A., Robinson S.T., West M.R., Mackey A.P., Yendiki A., Rowe M.L., Gabrieli J.D.E. (2018). Language Exposure Relates to Structural Neural Connectivity in Childhood. J. Neurosci..

[95] Hoff E., Burridge A., Ribot K.M., Giguere D. (2018). Language Specificity in the Relation of Maternal Education to Bilingual Children’s Vocabulary Growth. Dev. Psychol..

[96] Hoff-Ginsberg E. (1991). Mother-Child Conversation in Different Social Classes and Communicative Settings. Child Dev..

[97] Hoff E., Core C., Place S., Rumiche R., Señor M., Parra M. (2012). Dual Language Exposure and Early Bilingual Development. J. Child Lang..

[98] Schneider J.M., McIlvain G., Johnson C.L. (2022). Mechanical Properties of the Developing Brain Are Associated with Language Input and Vocabulary Outcome. Dev. Neuropsychol..

[99] Raizada R.D.S., Richards T.L., Meltzoff A., Kuhl P.K. (2008). Socioeconomic Status Predicts Hemispheric Specialisation of the Left Inferior Frontal Gyrus in Young Children. NeuroImage.

[100] Brito N.H., Troller-Renfree S.V., Leon-Santos A., Isler J.R., Fifer W.P., Noble K.G. (2020). Associations among the Home Language Environment and Neural Activity during Infancy. Dev. Cogn. Neurosci..

[101] Pierce L.J., Reilly E., Nelson C.A. (2021). Associations between Maternal Stress, Early Language Behaviors, and Infant Electroencephalography during the First Year of Life. J. Child Lang..

[102] Merz E.C., Wiltshire C.A., Noble K.G. (2019). Socioeconomic Inequality and the Developing Brain: Spotlight on Language and Executive Function. Child Dev. Perspect..

[103] Rosen M.L., Sheridan M.A., Sambrook K.A., Meltzoff A.N., McLaughlin K.A. (2018). Socioeconomic Disparities in Academic Achievement: A Multi-Modal Investigation of Neural Mechanisms in Children and Adolescents. NeuroImage.

[104] Noble K.G., Giebler M.A. (2020). The Neuroscience of Socioeconomic Inequality. Curr. Opin. Behav. Sci..

[105] NIMH Data Archive—Data Dictionary: Data Structure. https://nda.nih.gov/data_structure.html?short_name=inr01.

[106] Merz E.C., Desai P.M., Maskus E.A., Melvin S.A., Rehman R., Torres S.D., Meyer J., He X., Noble K.G. (2019). Socioeconomic Disparities in Chronic Physiologic Stress Are Associated with Brain Structure in Children. Biol. Psychiatry.

[107] Troller-Renfree S.V., Costanzo M.A., Duncan G.J., Magnuson K., Gennetian L.A., Yoshikawa H., Halpern-Meekin S., Fox N.A., Noble K.G. (2022). The Impact of a Poverty Reduction Intervention on Infant Brain Activity. Proc. Natl. Acad. Sci. USA.

[108] Brooks-Gunn J., Duncan G.J. (1997). The Effects of Poverty on Children. Future Child..

[109] McDonald C.R., Weckman A.M., Wright J.K., Conroy A.L., Kain K.C. (2022). Developmental Origins of Disease Highlight the Immediate Need for Expanded Access to Comprehensive Prenatal Care. Front. Public Health.

[110] Lautarescu A., Craig M.C., Glover V., Clow A., Smyth N. (2020). Chapter Two—Prenatal Stress: Effects on Fetal and Child Brain Development. International Review of Neurobiology.

[111] Conger R.D., Wallace L.E., Sun Y., Simons R.L., McLoyd V.C., Brody G.H. (2002). Economic Pressure in African American Families: A Replication and Extension of the Family Stress Model. Dev. Psychol..

[112] Conger R.D., Elder G.H. (1994). Families in Troubled Times: Adapting to Change in Rural America. Social Institutions and Social Change.

[113] Hobkirk A.L., Krebs N.M., Muscat J.E. (2018). Income as a Moderator of Psychological Stress and Nicotine Dependence among Adult Smokers. Addict. Behav..

[114] Luby J., Belden A., Botteron K., Marrus N., Harms M.P., Babb C., Nishino T., Barch D. (2013). The Effects of Poverty on Childhood Brain Development: The Mediating Effect of Caregiving and Stressful Life Events. JAMA Pediatr..

[115] Schulz A.J., Israel B.A., Zenk S.N., Parker E.A., Lichtenstein R., Shellman-Weir S., Ab L.K. (2006). Psychosocial Stress and Social Support as Mediators of Relationships between Income, Length of Residence and Depressive Symptoms among African American Women on Detroit’s Eastside. Soc. Sci. Med..

[116] Attar B.K., Guerra N.G., Tolan P.H. (1994). Neighborhood Disadvantage, Stressful Life Events and Adjustments in Urban Elementary-School Children. J. Clin. Child Psychol..

[117] Britt C.L. (1994). Crime and Unemployment among Youths in the United States, 1958–1990. Am. J. Econ. Sociol..

[118] Evans G.W. (2004). The Environment of Childhood Poverty. Am. Psychol..

[119] Santiago C.D., Wadsworth M.E., Stump J. (2011). Socioeconomic Status, Neighborhood Disadvantage, and Poverty-Related Stress: Prospective Effects on Psychological Syndromes among Diverse Low-Income Families. J. Econ. Psychol..

[120] Evans G.W., Wells N.M., Moch A. (2003). Housing and Mental Health: A Review of the Evidence and a Methodological and Conceptual Critique. J. Soc. Issues.

[121] Evans J., Benefield P. (2001). Systematic Reviews of Educational Research: Does the Medical Model Fit?. Br. Educ. Res. J..

[122] Poudel S., Denicola-Prechtl K., Nelson J.A., Behboudi M.H., Benitez-Barrera C., Castro S., Maguire M.J. (2024). Rethinking Household Size and Children’s Language Environment. Dev. Psychol..

[123] Benner A.D., Kim S.Y. (2010). Understanding Chinese American Adolescents’ Developmental Outcomes: Insights from the Family Stress Model. J. Res. Adolesc..

[124] Linver M.R., Brooks-Gunn J., Kohen D.E. (2002). Family Processes as Pathways from Income to Young Children’s Development. Dev. Psychol..

[125] Neppl T.K., Senia J.M., Donnellan M.B. (2016). The Effects of Economic Hardship: Testing the Family Stress Model over Time. J. Fam. Psychol..

[126] Lupien S.J., Maheu F., Tu M., Fiocco A., Schramek T.E. (2007). The Effects of Stress and Stress Hormones on Human Cognition: Implications for the Field of Brain and Cognition. Brain Cogn..

[127] Shonkoff J.P., Garner A.S., Siegel B.S., Dobbins M.I., Earls M.F., Garner A.S., McGuinn L., Pascoe J., Wood D.L., Committee on Psychosocial Aspects of Child and Family Health, Committee on Early Childhood, Adoption, and Dependent Care, and Section on Developmental and Behavioral Pediatrics (2012). The Lifelong Effects of Early Childhood Adversity and Toxic Stress. Pediatrics.

[128] Kim P., Evans G.W., Angstadt M., Ho S.S., Sripada C.S., Swain J.E., Liberzon I., Phan K.L. (2013). Effects of Childhood Poverty and Chronic Stress on Emotion Regulatory Brain Function in Adulthood. Proc. Natl. Acad. Sci. USA.

[129] McEwen B.S. (2010). Stress, Sex, and Neural Adaptation to a Changing Environment: Mechanisms of Neuronal Remodeling. Ann. N. Y. Acad. Sci..

[130] Wager T.D., Sylvester C.-Y.C., Lacey S.C., Nee D.E., Franklin M., Jonides J. (2005). Common and Unique Components of Response Inhibition Revealed by fMRI. NeuroImage.

[131] Hanson J.L., Chandra A., Wolfe B.L., Pollak S.D. (2011). Association between Income and the Hippocampus. PLoS ONE.

[132] Biegler R., McGregor A., Krebs J.R., Healy S.D. (2001). A Larger Hippocampus Is Associated with Longer-Lasting Spatial Memory. Proc. Natl. Acad. Sci. USA.

[133] Gianaros P.J., Jennings J.R., Sheu L.K., Greer P.J., Kuller L.H., Matthews K.A. (2007). Prospective Reports of Chronic Life Stress Predict Decreased Grey Matter Volume in the Hippocampus. NeuroImage.

[134] Piccolo L.R., Noble K.G., Pediatric Imaging, Neurocognition, and Genetics Study (2018). Perceived Stress Is Associated with Smaller Hippocampal Volume in Adolescence. Psychophysiology.

[135] McEwen B.S. (2000). Effects of Adverse Experiences for Brain Structure and Function. Biol. Psychiatry.

[136] Papagni S.A., Benetti S., Arulanantham S., McCrory E., McGuire P., Mechelli A. (2011). Effects of Stressful Life Events on Human Brain Structure: A Longitudinal Voxel-Based Morphometry Study. Stress.

[137] Radley J.J., Morrison J.H. (2005). Repeated Stress and Structural Plasticity in the Brain. Ageing Res. Rev..

[138] Cisler J.M., James G.A., Tripathi S., Mletzko T., Heim C., Hu X.P., Mayberg H.S., Nemeroff C.B., Kilts C.D. (2013). Differential Functional Connectivity within an Emotion Regulation Neural Network among Individuals Resilient and Susceptible to the Depressogenic Effects of Early Life Stress. Psychol. Med..

[139] Philip N.S., Valentine T.R., Sweet L.H., Tyrka A.R., Price L.H., Carpenter L.L. (2014). Early Life Stress Impacts Dorsolateral Prefrontal Cortex Functional Connectivity in Healthy Adults: Informing Future Studies of Antidepressant Treatments. J. Psychiatr. Res..

[140] Corning W.C., Steffy R.A., Anderson E., Bowers P. (1986). EEG “Maturational Lag” Profiles: Follow-up Analyses. J. Abnorm. Child Psychol..

[141] Harmony T., Hinojosa G., Marosi E., Becker J., Rodriguez M., Reyes A., Rocha C. (1990). Correlation between Eeg Spectral Parameters and an Educational Evaluation. Int. J. Neurosci..

[142] McLaughlin K.A., Fox N.A., Zeanah C.H., Sheridan M.A., Marshall P., Nelson C.A. (2010). Delayed Maturation in Brain Electrical Activity Partially Explains the Association between Early Environmental Deprivation and Symptoms of Attention-Deficit/Hyperactivity Disorder. Biol. Psychiatry.

[143] Pierce L.J., Thompson B.L., Gharib A., Schlueter L., Reilly E., Valdes V., Roberts S., Conroy K., Levitt P., Nelson C.A. (2019). Association of Perceived Maternal Stress during the Perinatal Period with Electroencephalography Patterns in 2-Month-Old Infants. JAMA Pediatr..

[144] Baumgartl H., Fezer E., Buettner R. Two-Level Classification of Chronic Stress Using Machine Learning on Resting-State EEG Recordings. Proceedings of the 25th Americas Conference on Information Systems.

[145] Saeed S.M.U., Anwar S.M., Majid M., Bhatti A.M. Psychological Stress Measurement Using Low Cost Single Channel EEG Headset. Proceedings of the 2015 IEEE International Symposium on Signal Processing and Information Technology (ISSPIT).

[146] Vanhollebeke G., Kappen M., De Raedt R., Baeken C., van Mierlo P., Vanderhasselt M.-A. (2023). Effects of Acute Psychosocial Stress on Source Level EEG Power and Functional Connectivity Measures. Sci. Rep..

[147] Tan E., Tang A., Debnath R., Humphreys K.L., Zeanah C.H., Nelson C.A., Fox N.A. (2023). Resting Brain Activity in Early Childhood Predicts IQ at 18 Years. Dev. Cogn. Neurosci..

[148] Ellwood-Lowe M.E., Sacchet M.D., Gotlib I.H. (2016). The Application of Neuroimaging to Social Inequity and Language Disparity: A Cautionary Examination. Dev. Cogn. Neurosci..

[149] Bester S., Malan-Van Rooyen M., Wright J.D. (2015). Emotional Development, Effects of Parenting and Family Structure On. International Encyclopedia of the Social & Behavioral Sciences.

[150] Ruggles S., Heggeness M. (2008). Intergenerational Coresidence in Developing Countries. Popul. Dev. Rev..

[151] Cohn D., Horowitz J.M., Minkin R., Fry R., Hurst K. (2022). The Demographics of Multigenerational Households. Pew Research Center’s Social & Demographic Trends Project.

[152] Sperry D.E., Sperry L.L., Miller P.J. (2019). Reexamining the Verbal Environments of Children from Different Socioeconomic Backgrounds. Child Dev..

[153] Dailey S., Bergelson E. (2022). Language Input to Infants of Different Socioeconomic Statuses: A Quantitative Meta-Analysis. Dev. Sci..

[154] Gilkerson J., Richards J.A., Warren S.F., Montgomery J.K., Greenwood C.R., Kimbrough O.D., Hansen J.H.L., Paul T.D. (2017). Mapping the Early Language Environment Using All-Day Recordings and Automated Analysis. Am. J. Speech-Lang. Pathol..

[155] Keene J.R., Batson C.D. (2010). Under One Roof: A Review of Research on Intergenerational Coresidence and Multigenerational Households in the United States. Sociol. Compass.

[156] Romeo R.R., Leonard J.A., Grotzinger H.M., Robinson S.T., Takada M.E., Mackey A.P., Scherer E., Rowe M.L., West M.R., Gabrieli J.D.E. (2021). Neuroplasticity Associated with Changes in Conversational Turn-Taking Following a Family-Based Intervention. Dev. Cogn. Neurosci..

[157] Rowe M.L., Denmark N., Harden B.J., Stapleton L.M. (2016). The Role of Parent Education and Parenting Knowledge in Children’s Language and Literacy Skills among White, Black, and Latino Families. Infant Child Dev..

[158] Gonzalez J.E., Acosta S., Davis H., Pollard-Durodola S., Saenz L., Soares D., Resendez N., Zhu L. (2017). Latino Maternal Literacy Beliefs and Practices Mediating Socioeconomic Status and Maternal Education Effects in Predicting Child Receptive Vocabulary. Early Educ. Dev..

[159] Suskind D.L., Leung C.Y.Y., Webber R.J., Hundertmark A.C., Leffel K.R., Fuenmayor Rivas I.E., Grobman W.A. (2018). Educating Parents about Infant Language Development: A Randomized Controlled Trial. Clin. Pediatr..

[160] Hammer C.S., Weiss A.L. (2000). African American Mothers’ Views of Their Infants’ Language Development and Language-Learning Environment. Am. J. Speech-Lang. Pathol..

[161] Luo R., Song L., Villacis C., Santiago-Bonilla G. (2021). Parental Beliefs and Knowledge, Children’s Home Language Experiences, and School Readiness: The Dual Language Perspective. Front. Psychol..

[162] Draper C.E., Barnett L.M., Cook C.J., Cuartas J.A., Howard S.J., McCoy D.C., Merkley R., Molano A., Maldonado-Carreño C., Obradović J. (2023). Publishing Child Development Research from around the World: An Unfair Playing Field Resulting in Most of the World’s Child Population under-Represented in Research. Infant Child Dev..

[163] Nielsen M., Haun D., Kärtner J., Legare C.H. (2017). The Persistent Sampling Bias in Developmental Psychology: A Call to Action. J. Exp. Child Psychol..

[164] Jackson M.I., Kiernan K., McLanahan S. (2017). Maternal education, changing family circumstances, and children’s skill development in the United States and UK. ANN. Am. Acad. Polit. Soc. Sci..

[165] Jeong J., Kim R., Subramanian S.V. (2018). How consistent are associations between maternal and paternal education and child growth and development outcomes across 39 low-income and middle-income countries?. J. Epidemiol. Community Health.

[166] Qi D., Wu Y. (2020). Family’s social economic status and child educational outcomes in China: The mediating effects of parenting practices and children’s learning attitudes. Child. Youth Serv. Rev..

[167] Rowe M.L., Weisleder A. (2020). Language Development in Context. Annu. Rev. Dev. Psychol..

[168] Casillas M., Brown P., Levinson S.C. (2020). Early Language Experience in a Tseltal Mayan Village. Child Dev..

[169] Cristia A. (2023). A Systematic Review Suggests Marked Differences in the Prevalence of Infant-Directed Vocalization across Groups of Populations. Dev. Sci..

[170] Cristia A., Dupoux E., Gurven M., Stieglitz J. (2019). Child-Directed Speech Is Infrequent in a Forager-Farmer Population: A Time Allocation Study. Child Dev..

[171] Shneidman L.A., Goldin-Meadow S. (2012). Language Input and Acquisition in a Mayan Village: How Important Is Directed Speech?. Dev. Sci..

[172] Byers-Heinlein K., Esposito A.G., Winsler A., Marian V., Castro D.C., Luk G. (2019). The Case for Measuring and Reporting Bilingualism in Developmental Research. Collabra Psychol..

[173] Tucker G.R. (2001). A Global Perspective on Bilingualism and Bilingual Education. Georgetown University Round Table on Languages and Linguistics 1999.

[174] Surrain S., Luk G. (2019). Describing Bilinguals: A Systematic Review of Labels and Descriptions Used in the Literature between 2005–2015. Biling. Lang. Cogn..

[175] KIDS COUNT Data Center Children Who Speak a Language Other than English at Home. https://datacenter.aecf.org/data/tables/81-children-who-speak-a-language-other-than-english-at-home.

[176] Katsiaficas M.P., O’Toole A. Caitlin Dual Language Learners: A National Demographic and Policy Profile. https://www.migrationpolicy.org/research/dual-language-learners-national-demographic-and-policy-profile.

[177] Espinosa L.M., LaForett D.R., Burchinal M., Winsler A., Tien H.-C., Peisner-Feinberg E.S., Castro D.C. (2017). Child Care Experiences among Dual Language Learners in the United States: Analyses of the Early Childhood Longitudinal Study–Birth Cohort. AERA Open.

[178] Rojas R., Iglesias A., Bunta F., Goldstein B., Goldenberg C., Reese L. (2016). Interlocutor Differential Effects on the Expressive Language Skills of Spanish-Speaking English Learners. Int. J. Speech-Lang. Pathol..

[179] Duncan T.S., Paradis J. (2020). Home Language Environment and Children’s Second Language Acquisition: The Special Status of Input from Older Siblings. J. Child Lang..

[180] Verdon S., McLeod S., Winsler A. (2014). Language Maintenance and Loss in a Population Study of Young Australian Children. Early Child. Res. Q..

[181] DeLuca V., Rothman J., Bialystok E., Pliatsikas C. (2020). Duration and Extent of Bilingual Experience Modulate Neurocognitive Outcomes. NeuroImage.

[182] Li L., Abutalebi J., Zou L., Yan X., Liu L., Feng X., Wang R., Guo T., Ding G. (2015). Bilingualism Alters Brain Functional Connectivity between “Control” Regions and “Language” Regions: Evidence from Bimodal Bilinguals. Neuropsychologia.

[183] Pliatsikas C., DeLuca V., Voits T. (2020). The Many Shades of Bilingualism: Language Experiences Modulate Adaptations in Brain Structure. Lang. Learn..

[184] Dash T., Berroir P., Joanette Y., Ansaldo A.I. (2019). Alerting, Orienting, and Executive Control: The Effect of Bilingualism and Age on the Subcomponents of Attention. Front. Neurol..

[185] Berken J.A., Chai X., Chen J.-K., Gracco V.L., Klein D. (2016). Effects of Early and Late Bilingualism on Resting-State Functional Connectivity. J. Neurosci..

[186] Grady C.L., Luk G., Craik F.I.M., Bialystok E. (2015). Brain Network Activity in Monolingual and Bilingual Older Adults. Neuropsychologia.

[187] Gullifer J.W., Chai X.J., Whitford V., Pivneva I., Baum S., Klein D., Titone D. (2018). Bilingual Experience and Resting-State Brain Connectivity: Impacts of L2 Age of Acquisition and Social Diversity of Language Use on Control Networks. Neuropsychologia.

[188] Sun X., Li L., Ding G., Wang R., Li P. (2019). Effects of Language Proficiency on Cognitive Control: Evidence from Resting-State Functional Connectivity. Neuropsychologia.

[189] Yamasaki B.L., Stocco A., Liu A.S., Prat C.S. (2019). Effects of Bilingual Language Experience on Basal Ganglia Computations: A Dynamic Causal Modeling Test of the Conditional Routing Model. Brain Lang..

[190] Bialystok E., Craik F.I.M., Luk G. (2012). Bilingualism: Consequences for Mind and Brain. Trends Cogn. Sci..

[191] Berken J.A., Gracco V.L., Chen J.-K., Watkins K.E., Baum S., Callahan M., Klein D. (2015). Neural Activation in Speech Production and Reading Aloud in Native and Non-Native Languages. NeuroImage.

[192] Jasinska K.K., Petitto L.A. (2013). How Age of Bilingual Exposure Can Change the Neural Systems for Language in the Developing Brain: A Functional near Infrared Spectroscopy Investigation of Syntactic Processing in Monolingual and Bilingual Children. Dev. Cogn. Neurosci..

[193] Lauharatanahirun N., Maciejewski D., Holmes C., Deater-Deckard K., Kim-Spoon J., King-Casas B. (2018). Neural Correlates of Risk Processing among Adolescents: Influences of Parental Monitoring and Household Chaos. Child Dev..

[194] Andrews J.L., Ahmed S.P., Blakemore S.-J. (2021). Navigating the Social Environment in Adolescence: The Role of Social Brain Development. Biol. Psychiatry.

[195] Iwinski S., Donovan S.M., Fiese B., Bost K. (2021). The Impact of Household Chaos and Dietary Intake on Executive Function in Young Children. Nutrients.

[196] Razza R.A., Martin A., Brooks-Gunn J. (2012). The Implications of Early Attentional Regulation for School Success among Low-Income Children. J. Appl. Dev. Psychol..

[197] Blair C., Raver C.C. (2016). Poverty, Stress, and Brain Development: New Directions for Prevention and Intervention. Acad. Pediatr..

[198] Lecheile B.M., Spinrad T.L., Xu X., Lopez J., Eisenberg N. (2020). Longitudinal Relations among Household Chaos, SES, and Effortful Control in the Prediction of Language Skills in Early Childhood. Dev. Psychol..

[199] Vernon-Feagans L., Willoughby M., Garrett-Peters P. (2016). Predictors of Behavioral Regulation in Kindergarten: Household Chaos, Parenting, and Early Executive Functions. Dev. Psychol..

[200] Coley R.L., Lynch A.D., Kull M. (2015). Early Exposure to Environmental Chaos and Children’s Physical and Mental Health. Early Child. Res. Q..

[201] Deater-Deckard K., Sewell M.D., Petrill S.A., Thompson L.A. (2010). Maternal Working Memory and Reactive Negativity in Parenting. Psychol. Sci..

[202] Xu J., Liu X., Li Q., Goldblatt R., Qin W., Liu F., Chu C., Luo Q., Ing A., Guo L. (2022). Global Urbanicity Is Associated with Brain and Behaviour in Young People. Nat. Hum. Behav..

[203] Evans G.W., Gonnella C., Marcynyszyn L.A., Gentile L., Salpekar N. (2005). The Role of Chaos in Poverty and Children’s Socioemotional Adjustment. Psychol. Sci..

[204] Essen J., Fogelman K., Head J. (1978). Children’s Housing and Their Health and Physical Development. Child Care Health Dev..

[205] Evans G.W., Maxwell L.E., Hart B. (1999). Parental Language and Verbal Responsiveness to Children in Crowded Homes. Dev. Psychol..

[206] Evans G.W., Saegert S., Harris R. (2001). Residential Density and Psychological Health among Children in Low-Income Families. Environ. Behav..

[207] Havron N., Lovcevic I., Kee M.Z.L., Chen H., Chong Y.S., Daniel M., Broekman B.F.P., Tsuji S. (2022). The Effect of Older Sibling, Postnatal Maternal Stress, and Household Factors on Language Development in Two- to Four-Year-Old Children. Dev. Psychol..

[208] Klatte M., Bergstroem K., Lachmann T. (2013). Does Noise Affect Learning? A Short Review on Noise Effects on Cognitive Performance in Children. Front. Psychol..

[209] Kujala T., Brattico E. (2009). Detrimental Noise Effects on Brain’s Speech Functions. Biol. Psychol..

[210] Maxwell L.E., Evans G.W. (2000). The effects of noise on pre-school children’s pre-reading skills. J. Environ. Psychol..

[211] Foraster M., Esnaola M., López-Vicente M., Rivas I., Álvarez-Pedrerol M., Persavento C., Sebastian-Galles N., Pujol J., Dadvand P., Sunyer J. (2022). Exposure to Road Traffic Noise and Cognitive Development in Schoolchildren in Barcelona, Spain: A Population-Based Cohort Study. PLoS Med..

[212] Werchan D.M., Brandes-Aitken A., Brito N.H. (2022). Signal in the Noise: Dimensions of Predictability in the Home Auditory Environment Are Associated with Neurobehavioral Measures of Early Infant Sustained Attention. Dev. Psychobiol..

[213] Simon K.R., Merz E.C., He X., Noble K.G. (2022). Environmental Noise, Brain Structure, and Language Development in Children. Brain Lang..

[214] Martínez-Vilavella G., Pujol J., Blanco-Hinojo L., Deus J., Rivas I., Persavento C., Sunyer J., Foraster M. (2023). The Effects of Exposure to Road Traffic Noise at School on Central Auditory Pathway Functional Connectivity. Environ. Res..

[215] Cohen S., Krantz D.S., Evans G.W., Stokols D., Kelly S. (1981). Aircraft Noise and Children: Longitudinal and Cross-Sectional Evidence on Adaptation to Noise and the Effectiveness of Noise Abatement. J. Personal. Soc. Psychol..

[216] Jones P.C., Pendergast L.L., Schaefer B.A., Rasheed M., Svensen E., Scharf R., Shrestha R., Maphula A., Roshan R., Rasmussen Z. (2017). Measuring Home Environments across Cultures: Invariance of the HOME Scale across Eight International Sites from the MAL-ED Study. J. Sch. Psychol..

[217] Garcini L.M., Arredondo M.M., Berry O., Church J.A., Fryberg S., Thomason M.E., McLaughlin K.A. (2022). Increasing Diversity in Developmental Cognitive Neuroscience: A Roadmap for Increasing Representation in Pediatric Neuroimaging Research. Dev. Cogn. Neurosci..

[218] Polemiti E., Hese S., Schepanski K., Yuan J., Schumann G., environMENTAL Consortium (2023). How Does the Macroenvironment Influence Brain and Behaviour—A Review of Current Status and Future Perspectives. medRxiv.

[219] Choy T., Baker E., Stavropoulos K. (2022). Systemic Racism in EEG Research: Considerations and Potential Solutions. Affect. Sci..

[220] Ricard J.A., Parker T.C., Dhamala E., Kwasa J., Allsop A., Holmes A.J. (2023). Confronting Racially Exclusionary Practices in the Acquisition and Analyses of Neuroimaging Data. Nat. Neurosci..

[221] Louis C.C., Webster C.T., Gloe L.M., Moser J.S. (2022). Hair Me out: Highlighting Systematic Exclusion in Psychophysiological Methods and Recommendations to Increase Inclusion. Front. Hum. Neurosci..

[222] Gatzke-Kopp L.M. (2016). Diversity and Representation: Key Issues for Psychophysiological Science. Psychophysiology.

[223] Roberts S.O., Bareket-Shavit C., Dollins F.A., Goldie P.D., Mortenson E. (2020). Racial Inequality in Psychological Research: Trends of the Past and Recommendations for the Future. Perspect. Psychol. Sci..

[224] Fulvio J.M., Akinnola I., Postle B.R. (2021). Gender (Im)Balance in Citation Practices in Cognitive Neuroscience. J. Cogn. Neurosci..

[225] Taylor C.M., Pritschet L., Jacobs E.G. (2021). The Scientific Body of Knowledge—Whose Body Does It Serve? A Spotlight on Oral Contraceptives and Women’s Health Factors in Neuroimaging. Front. Neuroendocrinol..

[226] Goldfarb M.G., Brown D.R. (2022). Diversifying Participation: The Rarity of Reporting Racial Demographics in Neuroimaging Research. NeuroImage.

[227] Fisher J.A., Kalbaugh C.A. (2011). Challenging Assumptions About Minority Participation in US Clinical Research. Am. J. Public Health.

[228] Wendler D., Kington R., Madans J., Wye G.V., Christ-Schmidt H., Pratt L.A., Brawley O.W., Gross C.P., Emanuel E. (2005). Are Racial and Ethnic Minorities Less Willing to Participate in Health Research?. PLoS Med..

[229] Webb E.K., Etter J.A., Kwasa J.A. (2022). Addressing Racial and Phenotypic Bias in Human Neuroscience Methods. Nat. Neurosci..

[230] Long Shadows: The Black-White Gap in Multigenerational Poverty. https://www.brookings.edu/articles/long-shadows-the-black-white-gap-in-multigenerational-poverty/.

[231] Heard-Garris N., Boyd R., Kan K., Perez-Cardona L., Heard N.J., Johnson T.J. (2021). Structuring Poverty: How Racism Shapes Child Poverty and Child and Adolescent Health. Acad. Pediatr..

[232] Simmons A., Taylor E.K., Abdurokhmonova G., Romeo R.R. (2023). Developing Best Practices for Inclusion in Pediatric fNIRS Research: Equity for Participants with Afro-Textured Hair.

[233] Brown L., Rollock D., Foti D. (2023). Conducting Electroencephalography with Black Individuals: Barriers, Recommendations, and Impact on Generalizability. Policy Insights Behav. Brain Sci..

[234] Marx D.M., Goff P.A. (2005). Clearing the Air: The Effect of Experimenter Race on Target’s Test Performance and Subjective Experience. Br. J. Soc. Psychol..

[235] Thorson K.R., Mendes W.B., West T.V. (2020). Controlling the Uncontrolled: Are There Incidental Experimenter Effects on Physiologic Responding?. Psychophysiology.

[236] Bradford D.E., DeFalco A., Perkins E.R., Carbajal I., Kwasa J., Goodman F.R., Jackson F., Richardson L.N.S., Woodley N., Neuberger L. (2024). Whose Signals Are Being Amplified? Toward a More Equitable Clinical Psychophysiology. Clin. Psychol. Sci..

